# Soluble CD93 Levels in Patients with Acute Myocardial Infarction and Its Implication on Clinical Outcome

**DOI:** 10.1371/journal.pone.0096538

**Published:** 2014-05-06

**Authors:** Jong-Chan Youn, Hee Tae Yu, Jae-Won Jeon, Hye Sun Lee, Yangsoo Jang, Young Woo Park, Yong-Beom Park, Eui-Cheol Shin, Jong-Won Ha

**Affiliations:** 1 Division of Cardiology, Severance Cardiovascular Hospital, Yonsei University College of Medicine, Seoul, Republic of Korea; 2 Laboratory of Immunology and Infectious Diseases, Graduate School of Medical Science and Engineering, KAIST, Daejeon, Republic of Korea; 3 Therapeutic Antibody Research Center, Korea Research Institute of Bioscience and Biotechnology, Daejeon, Republic of Korea; 4 Department of Biostatistics, Yonsei University College of Medicine, Seoul, Republic of Korea; 5 Yonsei Cardiovascular Genome Center, Severance Cardiovascular Hospital, Yonsei University College of Medicine, Seoul, Republic of Korea; 6 Division of Rheumatology, Department of Internal Medicine, Yonsei University College of Medicine, Seoul, Republic of Korea; University of Louisville, United States of America

## Abstract

**Background:**

Inflammation plays a key role in the pathogenesis of acute myocardial infarction (MI). However, it is unclear whether marker of immune activation will provide prognostic information in these patients. We hypothesized that circulating levels of soluble CD93 (sCD93), a soluble form of transmembrane glycoprotein CD93, is increased in acute MI patients and its level would be associated with clinical outcomes in patients with acute MI.

**Methods:**

We measured circulating levels of sCD93 in 120 patients with acute MI (63±13 yrs, M∶F = 85∶35) and in 120 age, sex-matched control subjects. In patients with acute MI, clinical characteristics, echocardiographic and laboratory findings were assessed at the time of initial enrollment. The primary outcome was defined as all-cause and cardiovascular death.

**Results:**

Circulating sCD93 levels were significantly higher in patients with acute MI than in control subjects (552.1±293.7 vs. 429.8±114.2 ng/mL, p<0.0001). Upon *in vitro* inflammatory stimulation, increased CD93 shedding was demonstrated in acute MI patients but not in control subjects. During follow up period (median 208 days, 3-1058 days), the primary outcome occurred in 18 (15%) patients (9 cardiovascular deaths). Circulating levels of sCD93 were associated with all cause (p<0.0001) and cardiovascular (p<0.0001) mortality in patients with acute MI. Multivariate Cox regression analysis revealed that initial sCD93 level was found to be an independent predictor of all cause (p = 0.002) and cardiovascular mortality (p = 0.033) when controlled for age and left ventricular ejection fraction.

**Conclusions:**

Circulating levels of sCD93 are elevated in patients with acute MI and their levels were associated with adverse clinical outcomes.

## Introduction

Myocardial infarction (MI) is defined as a clinical event caused by coronary thrombosis, and subsequent myocardial ischemia in which there is evidence of myocardial injury or necrosis [1]. Two major causes of coronary thrombosis are plaque rupture and endothelial erosion [2], [3]. Recent studies have shown that inflammation plays a key role in the pathogenesis of both plaque rupture and endothelial erosion. Activated immune cells produce various inflammatory molecules and proteolytic enzymes that can weaken the fibrous cap and activate cells in the core, transforming the stable plaque into a vulnerable, unstable structure that can rupture, induce a thrombus, and elicit an acute myocardial infarction. Because inflammation plays a key role in the pathogenesis of acute MI, a relevant biomarker of immune activation may provide novel prognostic information in these patients.

CD93 is a 68 kDa transmembrane glycoprotein and is expressed in monocytes, leukocytes, and endothelial cells. CD93 is over-expressed upon inflammatory stimulation, and the soluble form (sCD93) is known to be increased in various inflammatory conditions [4]–[6]. It has been shown that sCD93 induces the differentiation of monocytes into macrophage-like cells that have increased phagocytic activities and enhanced cell adhesion, and it has been implicated in inflammation and inflammatory diseases such as rheumatoid arthritis [6].

A recent study demonstrated that CD93 is correlated with the risk of coronary artery disease (CAD) [7]–[9]. In a genetic replication study, van der Net et al. [8] reported that patients homozygous for the T-allele of the CD93 genetic polymorphism had a 26% increased risk of CAD compared with patients with at least one C-allele (p = 0.01). Significant associations between plasma sCD93 levels, premature MI, and the incidence of CAD were reported in two independent cohorts [9]. Also, it has been demonstrated that the minor allele of an single nucleotide polymorphism (SNP) in the 3′ untranslated region of the CD93 gene was associated with increases in both plasma sCD93 concentration (p = 0.03) and CD93 mRNA expression levels in peripheral blood mononuclear cells (p = 0.02). These data suggest that genetic polymorphisms of CD93 and circulating sCD93 levels are associated with CAD in cross-sectional studies.

However, the relationship between sCD93 and the prognosis of acute MI has never been investigated. Therefore, in the present study, we examined if the circulating level of sCD93 is increased in acute MI patients. In addition, to explore the possible underlying mechanism leading to increased sCD93, we compared the shedding of CD93 in monocytes between patients with acute MI and control subjects. Finally, we investigated the impact of circulating sCD93 levels on clinical outcomes in acute MI patients.

## Methods

### Study population

One hundred twenty patients who were diagnosed with acute MI at Severance Cardiovascular Hospital were enrolled. MI was defined as: 1) typical continuous chest pain for more than 30 min, 2) ST segment elevation of more than 0.1 mV in two or more successive leads or ST segment depression of more than 0.2 mV in two or more successive leads monitored by a standard 12 lead electrocardiogram, 3) either a rise of creatinine kinase-MB to greater than twice the normal level or an elevation of troponin T of ≥0.1 ng/mL. Patients underwent a complete physical examination, baseline electrocardiogram, and laboratory assessment at the time of initial enrollment. Serum sampling for the analysis of sCD93 was done at the morning following the admission. Patients with a history of overt chronic inflammatory diseases such as rheumatoid arthritis or autoimmune disease and/or those on anti-inflammatory medications were excluded from this study.

Age and gender-matched, healthy control subjects (n = 120) were randomly selected from volunteers who were voluntarily enrolled in the Yonsei Cardiovascular Genome Center. All control subjects had no symptoms or history of coronary artery disease, and had a normal baseline electrocardiogram.

### Ethics Statement

The study protocol was approved by the institutional review board (IRB) of Yonsei University College of Medicine and IRB number was 4-2004-0103. All subjects provided written informed consent to participate in this study.

### Sandwich enzyme-linked immunosorbent assay (ELISA) for sCD93

A 96-well micro assay plate was coated with mouse anti-human CD93 Ab (1 µg/mL, R&D systems, Minneapolis, MN) and then blocked with 3% skim milk. Human sera from either acute MI patients or healthy control subjects were added and incubated for 2 h. After washing, the plate was incubated with goat anti-mouse CD93 Ab (0.5 µg/mL, R&D systems) for 1 h. Subsequently, the plate was incubated with anti-goat IgG-horseradish peroxidase (HRP) for 1 h. After washing, the plate was colorimetrically developed with o-phenylenediamine dihydrochloride substrate (Sigma-Aldrich, St. Louis, MO), and absorbance was read with the VERSAmax tunable microplate reader (Molecular Devices, Toronto, Ontario, Canada). Recombinant human sCD93 (amino acid 24–552) fused with human IgG1 Fc was used as a standard molecule as previously described [6]. To avoid inter-observer variability, a single investigator blinded to the clinical variables and laboratory findings executed the sandwich ELISA experiments. Intra- and inter-assay coefficients of variation (CVs) were 1.7% and 6.3%, respectively.

### *In vitro* analysis for shedding of sCD93 from monocytes

Peripheral blood mononuclear cells were isolated from the venous blood of six acute MI patients or six age and gender-matched healthy control subjects by Ficoll-density gradient. Monocytes were isolated by CD14 magnetic beads (Miltenyi Biotec, Auburn, CA). The purity of the isolated monocytes was 92–97% in flow cytometry analysis with anti-CD14-APC Ab (BD Biosciences, San Jose, CA). The isolated monocytes were incubated with or without lipopolysaccharide (LPS) (5 µg/mL) for 24 h, and sCD93 levels were analyzed by sandwich ELISA in the culture supernatants.

### Statistical analysis

Continuous variables were presented as a mean±SD. Categorical variables were expressed as a percentage of the group total. Discrete variables were compared using the chi-squared method, and independent t-tests were used for continuous variables.

Regarding the cut-off value of the sCD93 for cumulative mortality, we decided cut-off point according to Youden index, for all cause mortality, natural logarithm (Ln) sCD93 6.48 (sensitivity 61.1% and specificity 87.3%) and for CV mortality, Ln sCD93 6.38 (sensitivity 88.9% and specificity 78.4%). The cumulative incidence of mortality was estimated using the Kaplan-Meier method. The significance of the curves was tested using the log-rank test.

Univariate and multivariate Cox regression analysis were performed to identify independent predictors of all-cause and cardiovascular death. Because of relatively low mortality rate (n = 18 of 120), three different models were made based on goodness of fit. Age, sCD93, and high sensitivity C-reactive protein (hsCRP) were the variables used for the best-fit model for all-cause mortality, while sCD93, age, and LVEF were those used in the best-fit model for cardiovascular mortality. To verify the effect of sCD93 in these models, a likelihood ratio (LR) chi-square test was done to compare the reduced model (model without sCD93) and the full model (model with sCD93).

Statistical analyses were performed using R package, version 3.0.2 (http://www.R-project.org) and statistical significance was defined as p<0.05.

## Results

### Circulating sCD93 levels in acute MI patients

Baseline clinical characteristics and laboratory findings are summarized in table 1 and detailed characteristics of acute MI patients are summarized in table 2. Circulating sCD93 levels were significantly higher in patients with acute MI than in control subjects (552.1±293.7 ng/mL vs. 429.8±114.2 ng/mL, p<0.0001). Patients with Non-ST elevation MI showed higher sCD93 levels when compared with those of patients with ST elevation MI (625.6±364.9 ng/mL vs. 491.9±203.0 ng/mL, p = 0.018). However, circulating sCD93 levels were not significantly different according to culprit artery (left main artery, left anterior descending artery, left circumflex artery and right coronary artery) or CAD extent. There was no significant correlation between sCD93 levels and hsCRP or peak cardiac enzyme in this study, showing a relatively different character of sCD93 as an inflammation marker. We also evaluated surface expression levels of CD93 in peripheral monocytes of 51 patients with acute MI whose peripheral blood mononuclear cells (PBMCs) were available. There was no significant correlation between surface expression level of CD93 in monocytes and the circulating level of sCD93. These results reveal that circulating sCD93 levels are selectively increased in patients with acute MI, regardless of the surface expression level of CD93 in peripheral monocytes.

**Table 1:** Clinical characteristics and laboratory findings of analyzed subjects.

	Acute MI (N = 120)	Control (N = 120)	p-value*
Age (years)	62.6±12.8	60.2±12.9	0.139
Male (%)	85 (70.8%)	85 (70.8%)	-
Hypertension (%)	73 (60.8%)	18 (15.0%)	<0.001
DM (%)	42 (35.0%)	5 (4.2%)	<0.001
Hemoglobin (g/dL)	13.4±2.3	14.3±1.6	0.001
T. chol (mg/dL)	170.5±52.2	197.0±30.8	<0.001
TG (mg/dL)	122.8±75.7	137.9±86.2	0.155
HDL (mg/dL)	39.0±10.6	49.0±12.9	<0.001
LDL (mg/dL)	101.7±39.9	120.4±.28.1	<0.001
FBS (mg/dL)	186.4±121.2	87.3±18.5	<0.001
BUN (mg/dL)	19.8±10.4	15.3±4.2	<0.001
Creatinine (mg/dL)	1.4±1.3	0.8±0.2	<0.001
Uric acid (mg/dL)	5.4±1.8	5.2±1.4	0.209
hsCRP (mg/L)	27.4±59.7	2.2±8.3	<0.001
sCD93 (ng/mL)	552.1±293.7	429.8±114.2	<0.001

Values are presented as n (%) or mean ± SD. DM, diabetes mellitus; T. chol, Total cholesterol; TG, triglyceride; HDL, high-density lipoprotein; LDL, low-density lipoprotein; FBS, fasting blood sugar; BUN, blood urea nitrogen; hsCRP, high sensitive C-reactive protein; sCD93, soluble CD93.

*p-value <0.05 is considered significant.

**Table 2:** Laboratory, angiographic, echocardiographic findings and treatment modalities of AMI patients.

Characteristics	Values
Laboratory findings	
Initial CK-MB (ng/mL)	31.5±62.0
Peak CK-MB (ng/mL)	143.7±165.0
Initial Troponin-T (ng/mL)	1.0±2.3
Peak Troponin-T (ng/mL)	1.7±3.1
NT-proBNP (pg/mL)	4676.1±9475.3
Culprit artery	
Left main	1 (0.8%)
LAD	50 (41.6%)
LCx	14 (11.7%)
RCA	46 (38.3%)
Angiography not done	9 (7.5%)
Echocardiographic data	
LVEF (%)	47.3±13.0
LVEDD (mm)	51.4±6.1
LVESD (mm)	38.1±7.4
LV mass index (g/m^2^)	106.0±26.6
LA Vol. index (ml/m^2^)	28.9±11.6
Types of treatment	
PCI	96 (80%)
CABG	7 (5.8%)
Medical treatment only	17 (14.2%)
Types of medication	
Aspirin	119 (99.2%)
Clopidogrel	114 (95.0%)
ACEi/ARB	102 (85.0%)
Beta-blocker	104 (86.7%)
Spironolactone	20 (16.7%)
CCB	23 (19.2%)
Statin	114 (95%)

Values are presented as a %, or mean ± SD; CK-MB, creatine kinase MB; NT-proBNP, N-terminal pro-brain natriuretic peptide; LAD, left anterior descending artery; LCx, left circumflex artery; RCA, right coronary artery; LVEF, left ventricular ejection fraction; LVEDD, left ventricular end diastolic dimension; LVESD, left ventricular end systolic dimension; LV mass index, left ventricular mass index; LA Vol. index, left atrial volume index; PCI, percutaneous coronary intervention; CABG, coronary artery bypass graft; ACEi, angiotensin converting enzyme inhibitor; ARB, angiotensin receptor blockade; CCB, calcium channel blocker.

### Increased shedding of sCD93 from monocytes in acute MI patients

**Figure 1:**
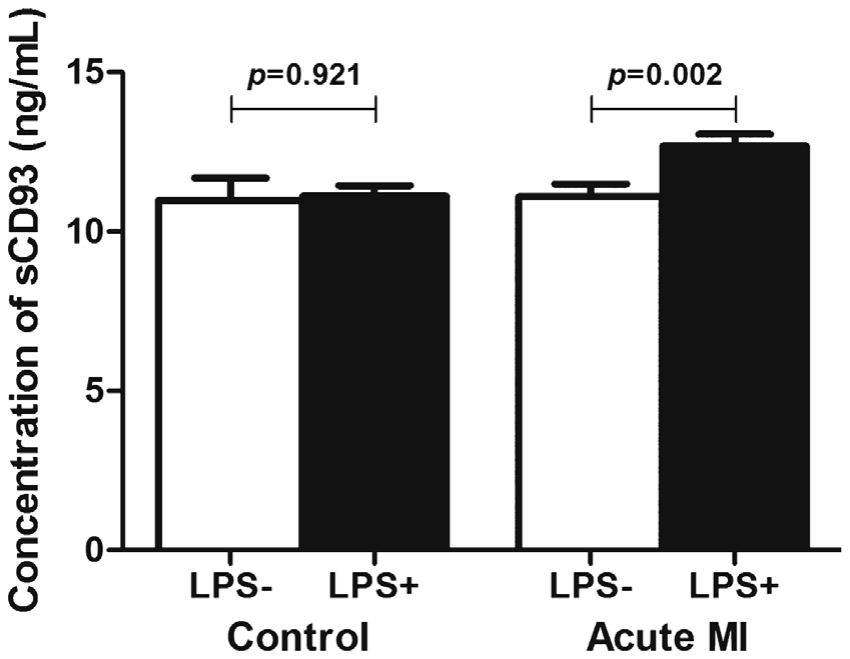
Increased amount of sCD93 in supernatant after LPS stimulation in acute MI patients.

To reveal the underlying mechanism of increased sCD93 in acute MI patients, we investigated shedding of CD93 in monocytes of acute MI patients and also in healthy control subjects with or without LPS. LPS is a well-known toll-like receptor stimulator which may mimic the inflammatory condition of acute MI. Without LPS stimulation, there was no difference between the level of sCD93 in monocytes of acute MI patients and control subjects. However, the amount of sCD93 in monocytes of acute MI patients with LPS stimulation was significantly higher than that of acute MI patients without LPS stimulation (12.7±0.9 vs. 11.1±1.0 ng/mL, respectively, p = 0.002, figure 1). There was no change of sCD93 in healthy control subjects with or without LPS stimulation 11.1±0.8 vs. 11.0±1.6 ng/mL, respectively, p = 0.921). Therefore, monocytes of acute MI patients showed increased susceptibility of CD93 shedding upon inflammatory stimulation when compared with that of healthy control subjects.

### Relationship of circulating sCD93 levels with clinical outcomes of acute MI patients

**Figure 2:**
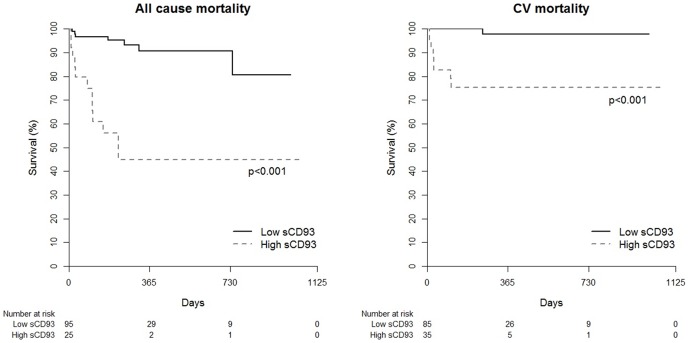
Cumulative Kaplan–Meier estimates of all cause and cardiovascular mortality according the circulating levels of sCD93.

We evaluated whether the increased level of sCD93 is associated with clinical outcomes in acute MI patients. During the follow-up period (median 208 days), the primary outcome, which was defined as all-cause and cardiovascular death, had occurred in 18 (15%) patients (9 cardiovascular deaths). When the patients with acute MI were divided by level of sCD93 according to Youden index, circulating levels of sCD93 were shown to be associated with all-cause (p<0.001) and cardiovascular (p<0.001) mortality (figure 2). Hosmer and Lemeshow analysis for all cause mortality (*P* = 0.2972) and CV mortality (*P* = 0.6841) showed acceptable calibration (*P*>0.05), which means good agreement between observed outcomes and predictions. Because of the relatively low rate of mortality (15%), three different models were made based on the goodness of fit. Age, sCD93, and hsCRP were the variables of the best fit model for all-cause mortality and sCD93, age, and LVEF were those of the best fit model for cardiovascular mortality. Multivariate Cox regression analysis revealed that the initial sCD93 level was an independent predictor of both all-cause (p = 0.005) and cardiovascular mortality (p = 0.033) when controlled for age and hsCRP or age and LVEF, respectively (table 3). The likelihood ratio chi-square test revealed that the sCD93 level was a significant variable in these models (Model 1, *P* = 0.003; Model 2, *P*<0.001; Model 3, *P*<0.001 for all-cause mortality, Model 1, *P* = 0.03; Model 2, *P* = 0.005; Model 3, *P*<0.001 for cardiovascular mortality).

**Table 3:** Univariate and multivariate Cox regression analysis for all-cause and cardiovascular mortality.

All-cause mortality	Univariate analysis		Multivariate analysis					
			Model 1 (−2 log L = 120.086)		Model 2 (−2 log L = 113.158)		Model 3 (−2 log L = 125.083)	
	HR (95% CI)	p-value*	HR (95% CI)	p-value*	HR (95% CI)	p-value*	HR (95% CI)	p-value*
Age	1.097 (1.042–1.154)	<0.001	1.122 (1.057–1.192)	<0.001	1.1 (1.033–1.171)	0.003	-	-
Hemoglobin	0.669 (0.547–0.818)	<0.001	-	-	-	-	-	-
Creatinine	1.263 (1.097–1.454)	<0.001	-	-	-	-	-	-
FBS	1.003 (1.001–1.006)	0.004	-	-	-	-	-	-
Homocysteine	1.089 (1.021–1.161)	0.009	-	-	-	-	-	-
hsCRP	1.005 (1.001–1.010)	0.022	-	-	1.429 (1.047–1.949)	0.024	1.446 (1.108–1.887)	0.007
LVEF	0.948 (0.909–0.988)	0.012	0.942 (0.900–0.986)	0.011	-	-	0.953 (0.911–0.998)	0.042
Ln sCD93	5.375 (2.415–11.965)	<0.001	5.051 (1.802–14.162)	0.002	3.935 (1.515–10.225)	0.005	4.828 (2.030–11.484)	<0.001

CV mortality	Univariate analysis		Multivariate analysis					
			Model 1 (−2 log L = 52.856)		Model 2 (−2 log L = 60.013)		Model 3 (−2 log L = 60.596)	
	HR (95% CI)	p-value*	HR (95% CI)	p-value*	HR (95% CI)	p-value*	HR (95% CI)	p-value*
Age	1.097 (1.018–1.182)	0.015	1.141 (1.043–1.248)	0.004	1.091(0.998–1.193)	0.054	-	-
Hemoglobin	0.639 (0.447–0.856)	0.003	-	-	-	-	-	-
Creatinine	1.326 (1.101–1.596)	0.003	-	-	-	-	-	-
FBS	1.003 (1.001–1.006)	0.013	-	-	-	-	-	-
Homocysteine	1.095 (1.006–1.193)	0.037	-	-	-	-	-	-
hsCRP	1.006 (1.001–1.012)	0.031	-	-	1.525 (0.971–2.395)	0.067	1.556 (1.065–2.272)	0.022
LVEF	0.896 (0.840–0.956)	0.001	0.891 (0.828–0.959)	0.002	-	-	0.91 (0.846–0.977)	0.01
Ln sCD93	7.904 (2.477–25.216)	<0.001	7.226 (1.176–44.393)	0.033	5.34 (1.386–20.574)	0.015	6.526 (1.605–26.531)	0.009

FBS, fasting blood sugar; hsCRP, high sensitive C-reactive protein; LVEF, left ventricular ejection fraction; sCD93, soluble CD93; HR, hazard ratio; −2 log L, −2 log likelihood.

*p-value <0.05 is considered significant.

## Discussion

In this study, we demonstrated that circulating levels of sCD93 are increased in patients with acute MI. In addition, monocytes isolated from acute MI patients show increased shedding of CD93 upon inflammatory stimulation, suggesting possible mechanism for the elevated sCD93 in these patients. Finally, we also showed that elevated levels of sCD93 were associated with adverse clinical outcomes in patients with acute MI.

The relationship between CD93 and CAD has been documented in previous genetic association studies [7], [8]. Mälarstig et al. [9] reported on the relationship between plasma levels of sCD93 and the risks of both premature MI and CAD in two independent cohorts. However, this study was a cross-sectional association study without prognostic implication. The present study is important because it is the first study to demonstrate the association between increased levels of sCD93 and clinical outcomes in patients with acute MI.

We observed an early divergence of the Kaplan-Meier curves shortly after the diagnosis of acute MI. Activation of chronic inflammation might be related with early adverse event after the acute MI, however the cause and effect relationship between inflammation and early adverse prognosis is not clear yet. There are no known relevant biomarkers of immune activation in atherosclerotic plaque rupture. hsCRP is the most extensively studied biomarker of inflammation in cardiovascular diseases. However, the prognostic value of hsCRP in patients with acute MI is controversial. Some studies have found that serum hsCRP predicts the risk of 30-day or long-term mortality after an MI [10], [11]. However, other reports have found no such relationship [12]. In this study, we have shown that the predictive prognostic value of sCD93 was independent of hsCRP in patients with acute MI.

Relationship between increased levels of sCD93 and adverse clinical outcomes in patients with acute MI might be explained by the underlying pathophysiology of atherosclerosis. Monocyte plays a key role from the beginning of the atherosclerotic plaque development to the final plaque rupture eliciting an acute MI. Circulating monocytes are recruited into the activated endothelium of artery and then differentiate into macrophage, which comprises major cellular component of atherosclerotic plaque. With the progression of atherosclerotic plaque, activated macrophage produces various pro-inflammatory cytokines, proteases, free oxygen radicals and vasoactive molecules that can destabilize the lesions. All these molecules might induce the activation and rupture of atherosclerotic plaque, thrombosis, and subsequently an acute MI [2], [3]. A recent study showed that sCD93 induced differentiation of monocytes to macrophage like cells, as evidenced by activated cell adhesion and increased phagocytic activities. In addition, this differentiation resulted in increased pro-inflammatory cytokine production [6]. The link between sCD93 and monocyte activation has been implicated in inflammation and circulating levels of sCD93 can be a maker of monocyte activation. In this study, we have demonstrated that circulating sCD93, novel monocyte inflammatory marker, are elevated in patients with acute MI and their levels were associated with adverse clinical outcomes.

The mechanism underlying elevated sCD93 levels in patients with acute MI might be an increased shedding of CD93 from monocytes in these patients. The lack of either a negative or positive correlation between CD93 expression in monocytes and the circulating level of sCD93 may suggest tight homeostatic control of CD93 expression in monocytes even during the inflammatory process. The fact that monocytes of acute MI patients showed increased susceptibility of CD93 shedding upon inflammatory stimulation suggest that differential response to inflammatory stimuli may underlie the reason why circulating sCD93 levels are increased in patients with acute MI. Detailed studies of the underlying mechanisms of and genetic influences on CD93 shedding in the physiological environment and its relevance to CAD need to be conducted.

The present study has several potential limitations. First, the differential power of circulating sCD93 concentration between patients with acute MI and control subjects is relatively low. Therefore, the cutoff value for the normal range of sCD93 cannot be determined in this study. This low differential power of circulating sCD93 may be related to the systemic origin of CD93 shedding. Increased level of sCD93 in patients with acute MI cannot be attributed to CAD-specific inflammation. Rather, it may represent an overall increase in the monocyte inflammatory burden. In addition, circulating sCD93 levels were studied only once at the acute stage without serial measurements.

Second, the primary outcome, which was defined as all-cause and cardiovascular death, had occurred in only 18 (15%) patients (nine cardiovascular deaths and nine non-cardiovascular deaths). This small number of primary outcomes prohibited the use of all significant variables in our multivariate analysis. Only two or three variables including sCD93 were able to be used. However, we used three different models based on goodness of fit to explore the clinical value and implications of sCD93 in patients with acute MI. Even after adjusting for the most well-known prognostic factors, age and LVEF, the initial sCD93 level was found to be an independent predictor of all-cause and cardiovascular mortality. Third, we could not measure other inflammation markers such as neopterin in this study. We only measured sCD93, hsCRP and cardiac enzymes. There was no significant correlation between sCD93 levels and hsCRP or peak cardiac enzyme in this study, showing a relatively different character of sCD93 as an inflammation marker. Finally, it is difficult to determine whether elevated sCD93 is the result or the cause of acute MI. Increased sCD93 levels may be related with the pathogenesis of acute MI, however further investigations are needed to identify the direct cause and effect relationship.

## Conclusions

In the present study, we have demonstrated that circulating levels of sCD93 are increased in patients with acute MI. Monocytes isolated from acute MI patients show increased shedding of CD93 upon inflammatory stimulation, suggesting possible mechanism for the elevated sCD93 in these patients. Finally, we also showed that elevated levels of sCD93 were associated with adverse clinical outcomes in patients with acute MI. Circulating sCD93 as a marker of monocyte inflammation might be considered for risk stratification in patients with acute MI and needs to be further studied to more completely understand the inflammatory pathophysiology of this disease process.
